# Droplet digital PCR quantification of miR-1290 as a circulating biomarker for pancreatic cancer

**DOI:** 10.1038/s41598-018-34597-z

**Published:** 2018-11-06

**Authors:** Francesca Tavano, Domenica Gioffreda, Maria R. Valvano, Orazio Palmieri, Matteo Tardio, Tiziana P. Latiano, Ada Piepoli, Evaristo Maiello, Felice Pirozzi, Angelo Andriulli

**Affiliations:** 1 Division of Gastroenterology and Research Laboratory, Fondazione IRCCS Casa Sollievo della Sofferenza, San Giovanni Rotondo (FG), 71013 Italy; 2 Department of Surgery, Fondazione IRCCS Casa Sollievo della Sofferenza, San Giovanni Rotondo (FG), 71013 Italy; 3 Department of Oncology, Fondazione IRCCS Casa Sollievo della Sofferenza, San Giovanni Rotondo (FG), 71013 Italy

## Abstract

Droplet digital PCR was used to validate miR-1290 as circulating biomarker for pancreatic cancer (PC). The diagnostic performance of miR-1290 was evaluate in 167 PC patients and 267 healthy subjects at clinical risk of developing the disease (HS). MiR-1290 plasma levels were compared to CA 19-9 determinations, and the combination of the two biomarkers was also taken into account. Plasma levels of miR-1290 were higher in PC patients compared to HS (p = 2.55 × 10^−16^). A similar trend was observed for CA 19-9 determinations (p = 1.03 × 10^−47^). ROC curve analysis revealed that miR-1290 in combination with CA 19-9 was effective for discriminating between PC patients and HS (AUC = 0.956, 95% CI = 0.933–0.979) than the two biomarkers tested alone (miR-1290: AUC = 0.734, 0.678–0.789; CA 19-9: AUC = 0.914, 0.877–0.951). The discriminating ability was higher when only PC patients with low or slightly increased CA 19-9 levels were compared with HS. MiR-1290 concentrations were not able to differentiate between PC patients with single or multiple risk factors for developing PC. Our data suggest that the absolute quantification of circulating miR-1290 levels does not allow to select patients at clinical risk of PC for entry into a surveillance program, and underline the methodological challenges still existing in utilizing circulating miRNAs as new promising biomarkers for PC.

## Introduction

Pancreatic cancer (PC) is the fourth most common cause of cancer related-death worldwide^1^. Despite progress in surgical and therapeutic management of PC patients, survival rates remain dismal^2–4^.

Currently, there are no screening guidelines for PC, and the carbohydrate antigen 19-9 (CA 19-9) is the only biomarker used for the diagnosis and follow-up of patients^5–7^. However, while CA 19-9 has acceptable sensitivity, it has poor specificity. In addition, it is not sufficiently accurate to detect early stage PC^8^. Indeed, this serological biomarker is often elevated in benign disorders of the bilio-pancreatic tract and in non-pancreatic gastrointestinal tumors, and, vice versa, not all patients with PC present with elevated CA 19-9 serum levels^9^. Thus, the identification of novel and more accurate non-invasive biomarkers holds promise for the detection of early PC and for the implementation of surveillance programs for populations at high-risk of PC.

MicroRNA (miRNA) expression in body fluids has been linked to different pathological conditions, including human cancers^10,11^. In particular, several studies based on microarray analysis have shown that miRNAs are significantly altered in the serum and plasma of PC patients compared with healthy subjects (HS). A meta-analysis of published studies has demonstrated that several circulating miRNAs have moderate diagnostic accuracy to discriminate PC patients from non-pancreatic cancer patients and HS^12–14^. Among the most relevant miRNAs, miR-1290 showed good diagnostic performance in distinguishing patients with PC and/or intraductal papillary mucinous neoplasm (IPMN) of the pancreas from HS^15^. The same biomarker was also highly correlated with the outcome in patients after pancreatic resection, with higher levels predicting poor survival. This latter finding has been attributed to the ability of this miRNA *in vitro* to influence both proliferation and invasiveness in PC cell lines^16^.

Although emerging evidence suggests circulating miRNAs are promising new biomarkers for human cancers, different experimental conditions, such as the collection and storage of biological samples, RNA purification methods, quantification and internal/external normalization, may all affect the results of the most commonly used quantification approach^17^. Previous methodological biases may also explain some of the heterogeneity seen in data between studies. Recently, droplet digital PCR (ddPCR) has been shown to overcome some previous analytical difficulties, allowing absolute quantification without the need for internal/external normalization: ddPCR shows higher sensitivity and reduced variability in detecting low-abundance miRNA, thus producing more reliable results than conventional quantitative PCR^18,19^.

In this study, we have used ddPCR to validate miR1290 as potential circulating biomarker for diagnosis of PC. To pursue this purpose we have quantified miR-1290 plasma levels in PC patients and HS, and have evaluated the diagnostic ability of the biomarker, in conjunction with serum CA 19-9, to distinguish between these two groups. The potential of circulating miR-1290 for predicting the long-term outcome of PC patients was also investigated.

## Results

The demographic features and associated risk factors for PC in the 167 PC patients and 267 HS are given in Table 1. As expected, the PC cohort included older patients (median age: PC 68 years vs. HC 60 years) and a higher prevalence of males (PC 51.5% vs. HS 43.3%). As to risk factors for PC, the percentage of subjects reported being smokers (PC 57.5%, HS 50.2%) and having a family history of cancer (PC 64.6%, HS 73.7%) was higher than those without the risk factor at issue in both the cohorts. These differences were not statistically different. The discovery cohort consisted of 93 PC patients and 150 HS, and the validation cohort of 74 PC patients and 117 HS. PC patients and HS included in the two cohorts did not differ regarding baseline features or the prevalence of risk factors (data shown in Supplementary Table 1).

**Table 1:** Demographics, distribution of clinical risk factors in healthy subjects (HS), patients with pancreatic cancer (PC), intraductal papillary mucinous neoplasm (IPMN), and chronic pancreatitis (CP), and baseline clinical-pathological features of patients with PC.

	HS (N = 267)	PC (N = 167)	IPMN (N = 19)	CP (N = 20)
Age, median (Q1–Q3)	60 (54–69)	68 (61–76)	73 (64–75.5)	54 (47–62.5)
<55 years, N (%)	70 (26.2)	23 (13.8)	1 (5.3)	11 (55)
≥55 years, N (%)	197 (73.8)	144 (86.2)	18 (94.7)	9 (45)
Gender, N (%)				
Male	116 (43.4)	86 (51.5)	8 (42.1)	14 (70)
Female	151 (56.6)	81 (48.5)	11 (57.9)	6 (30)
Body Mass Index, median (Q1–Q3)	27 (24–30)	25 (23–28)	27 (26.1–28.1)	23.2 (20.4–27)
≤30, N (%)	204 (76.4)	138 (85.2)	16 (84.2)	17 (85)
>30, N (%)	63 (23.6)	24 (14.8)	3 (15.8)	3 (15)
Smoker, N (%)				
No	133 (49.8)	71 (42.5)	13 (68.4)	4 (20)
Current/past	134 (50.2)	96 (57.5)	6 (31.6)	16 (80)
Alcohol abuse (≥3 drinks a day), N (%)				
No	250 (93.6)	151 (91)	7 (36.8)	5 (25)
Yes	17 (6.4)	15 (9)	12 (63.2)	15 (75)
Diabetes, N (%)				
No	230 (86.1)	102 (61.6)	13 (68.4)	12 (60)
Yes	37 (13.9)	65 (38.9)	6 (31.6)	8 (40)
Family history of cancer, N (%)				
No	70 (26.3)	57 (35.4)	10 (52.6)	6 (30)
Yes	196 (73.7)	104 (64.6)	9 (47.4)	14 (70)
Tumor location, N (%)				
Head		117 (71)		
Body		21 (13)		
Tail		27 (16)		
Pre-operative classification, N (%)				
Resectable		34 (20)		
Locally advanced		60 (36)		
Metastatic		73 (44)		
Surgery, N (%)				
No		138 (83)		
Yes		29 (17)		
Tumor stage, N (%)				
IB		1 (0.6)		
IIA		4 (2.5)		
IIB		25 (15.7)		
III		56 (35.2)		
IV		73 (45.9)		
Adjuvant therapy, N (%)				
No		63 (39)		
Yes		100 (61)		

The pathological characteristics of the 167 PC patients are shown in Table 1. The majority of these subjects had a cancer located in the head of the pancreas (71%), and presented with locally advanced or metastatic disease (36% and 44%, respectively). Of the 34 remaining patients with a cancer staged as resectable at the pre-operative work-up, 29 were considered fit for surgery and underwent pancreatic resection. The concentration of CA 19-9 tumor marker was below the normal value of 37 U/ml in 34/167 PC patients (20.4%); 8 out of these patients had a resectable disease at the diagnosis, while the remaining 26 patients were staged as locally advanced (n = 7) or metastatic PC (n = 19). More than two thirds of resected patients (81.1%) had cancer stage III or IV, accordingly to the American Joint Committee on Cancer (AJCC) tumor/node/metastasis (TNM) classification and staging system for PC^20^. Adjuvant chemotherapy was administered to 61% of patients, with the remaining individuals considered ineligible for chemotherapy. The characteristics of PC patients in the discovery and validation cohorts were similar (see Supplementary Table 1).

### Circulating miR-1290 and CA 19-9 levels in PC patients and HS

**Figure 1:**
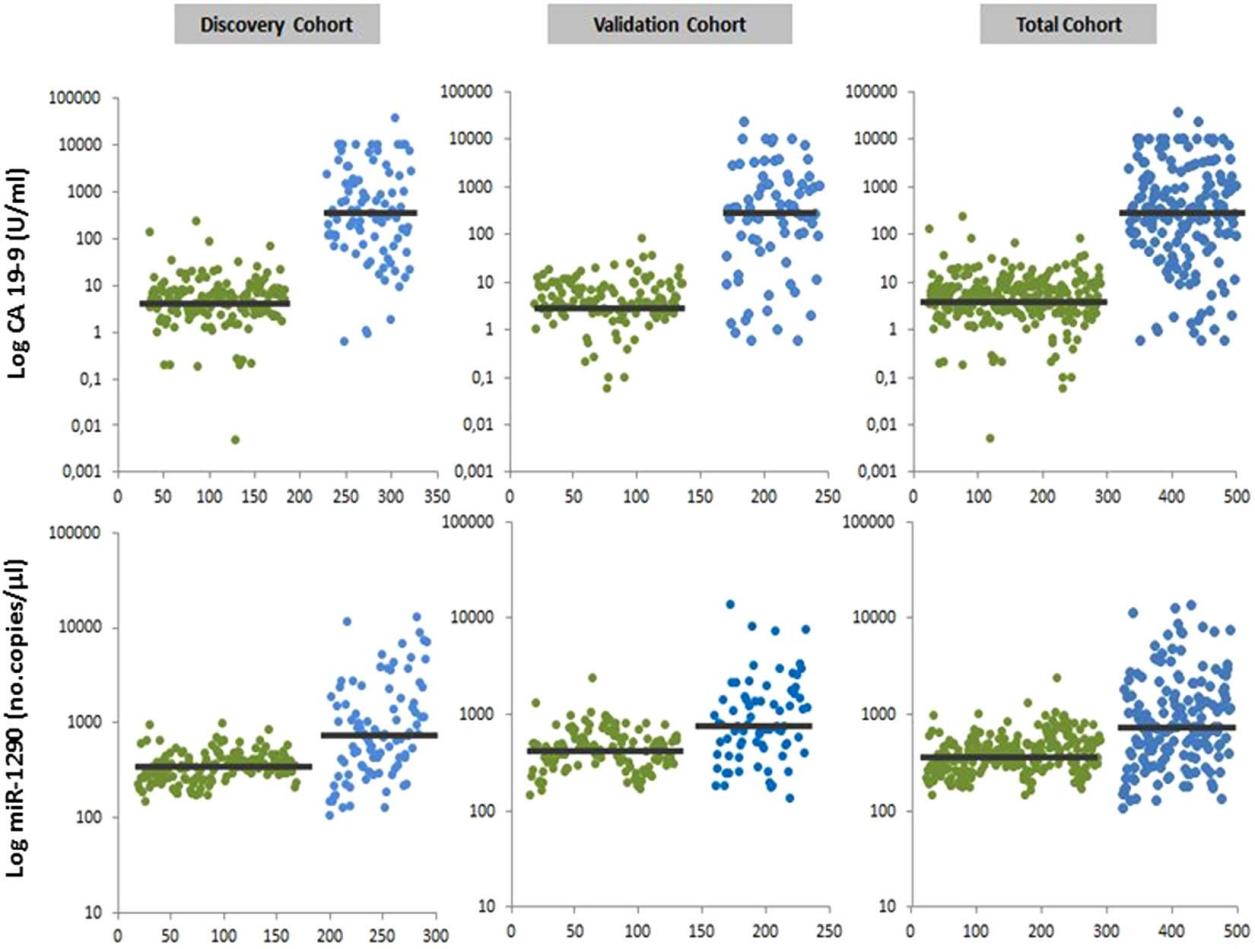
Absolute quantification of circulating levels of CA 19-9 and miR-1290 in healthy subjects (HS) and in patients with pancreatic cancer (PC). Each blot indicates the levels of CA 19-9 and miR-1290 tested using the ELISA assay and droplet digital PCR, respectively. The median levels in each HS and PC subgroup are indicated by the horizontal black bars. Values are reported in log scale (y-axis). HC: green dots; PC: blue dots

The distributions of miR-1290 and CA 19-9 levels in the study population are shown in Fig. 1. Median values for the two markers in the discovery and validation cohorts showed the same differences between PC patients and HC: median CA 19-9 levels were 337.5 U/ml (Q1–Q3 = 105.6–1821) vs. 5 U/ml (Q1–Q3 = 2.6–7.6) in the discovery cohort (p = 1.07 × 10^−31^), and 279.1 U/ml (Q1–Q3 = 44.2–1144) vs. 3.9 U/ml (Q1–Q3 = 2.3–8.8) in the validation cohort (p = 4.79 × 10^−18^); median miR-1290 levels were 716 no. copies/μl (Q1–Q3 = 348–1620) vs. 344 no. copies/μl (Q1–Q3 = 265.2–416) in the discovery cohort (p = 1.59 × 10^−10^), and 746 no. copies/μl (Q1–Q3 = 440–1452) vs. 424 no. copies/μl (Q1–Q3 = 300–576) in the validation cohort (p = 4.88 × 10^−7^).

In the combined cohort, median circulating CA 19-9 levels were 299 U/ml (Q1–Q3 = 70.1–1615) in PC patients and 4.3 U/ml (Q1–Q3 = 2.4–7.9) in HS (p = 1.03 × 10^−47^). Median miR-1290 levels were 744 no. copies/μl (Q1–Q3 = 380–1560) vs. 360 no. copies/μl (Q1–Q3 = 281.2–496) in the groups, respectively, indicating a significant difference (p = 2.55 × 10^−16^).

### The effect of haemolysis on miR-1290 plasma levels

The median miR-1290 levels were not statistically different in plasma samples with A_414_ > 0.2, compared to those with absorbance level below this cut-off level (HS: median = 340 no.copie/μl, Q1–Q3 = 260–484 vs median = 324 no.copies/μl, Q1–Q3 = 224–394; PC: median = 772 no.copie/μl, Q1–Q3 = 466–4986 vs median = 722 no.copies/μl, Q1–Q3 = 354–2897). Similarly, median miR-1290 levels did not differ significantly between samples having a value for the lipemia-independent hemolysis score above and below the established threshold levels (HS: median = 378 no.copie/μl, Q1–Q3 = 309.1–440 vs median = 326 no.copies/μl, Q1–Q3 = 232.3–422; PC: median = 852 no.copie/μl, Q1–Q3 = 453–5081 vs median = 664 no.copies/μl, Q1–Q3 = 356–2158). Accordingly to these results, all the samples were included in the following analyses.

### Diagnostic performance of plasma miR-1290 and serum CA 19-9 levels

**Figure 2:**
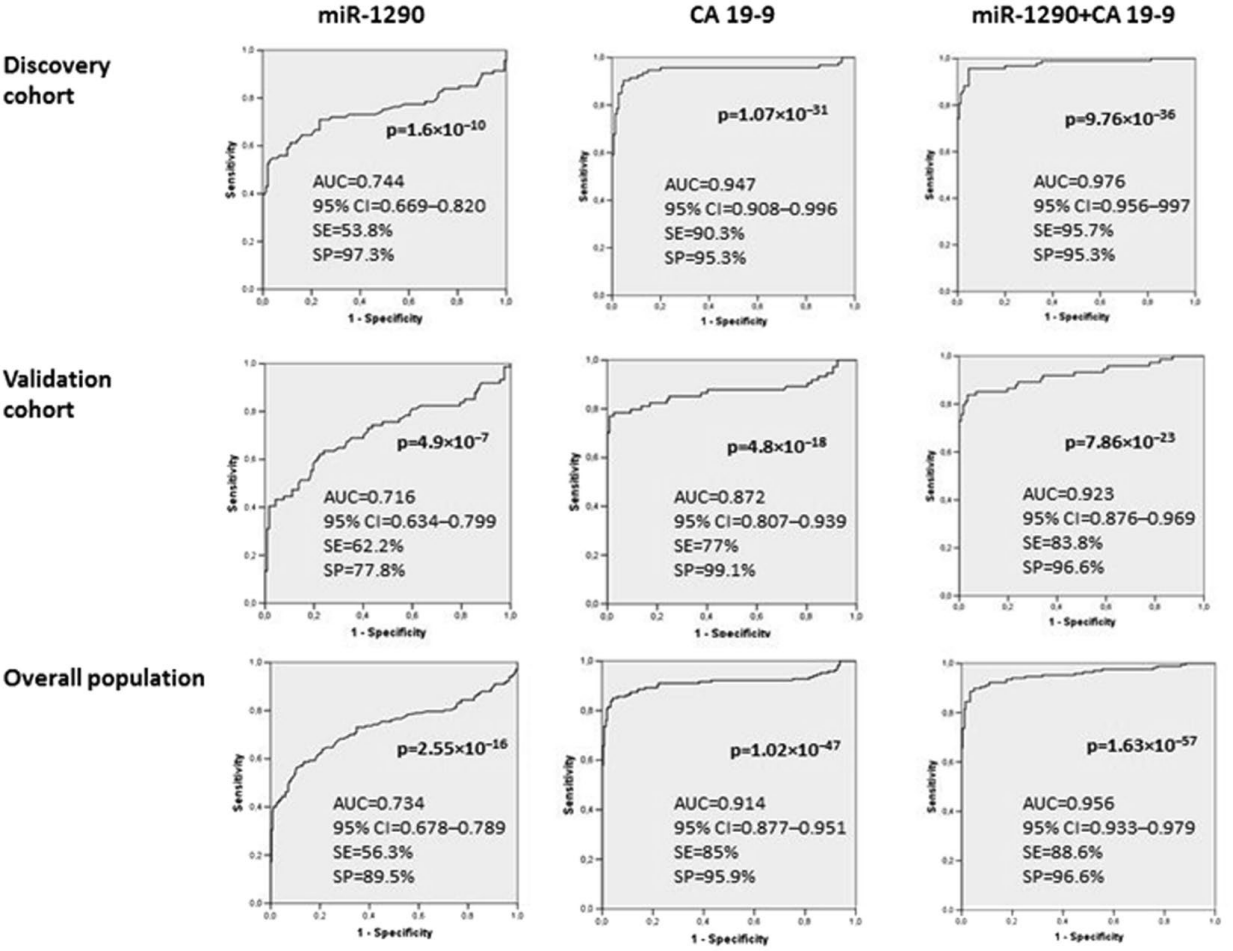
Receiver operating characteristics (ROC) curve analysis for discriminating healthy subjects (HS) from patients with pancreatic cancer (PC). The diagnostic performance of miR-1290 and CA 19-9 was tested independently and then in combination using a logistic regression model.

The ROC curves in Fig. 2 show the ability of the circulating levels of the two biomarkers to differentiate between PC patients and HS.

The cut-off values with the best discriminating ability in the discovery cohort (miR-1290 = 660 no. copies/μl; CA 19-9 = 21.5 U/ml) were in good agreement with those derived from the validation cohort (miR-1290 = 610 no. copies/μl; CA 19-9 = 36 U/ml). After the discovery and validation cohorts were combined, the best discriminating cut-off value was 662 no. copies/μl for miR-1290 (AUC of 0.734, 95% CI 0.678–0.789) which showed a sensitivity of 56.3% and a specificity of 89.5%. For the serum CA 19-9 levels, the best discriminating cut-off value was 21.7 U/ml (AUC of 0.914, 95% CI 0.877–0.951), with a sensitivity of 85% and a specificity of 95.9%. When the results of the two biomarkers were combined, the discriminatory ability improved compared with the performance of the CA 19-9 assay: AUC = 0.956 (95% CI 0.933–0.979), with a sensitivity of 88.6% and a specificity of 96.6%.

**Figure 3:**
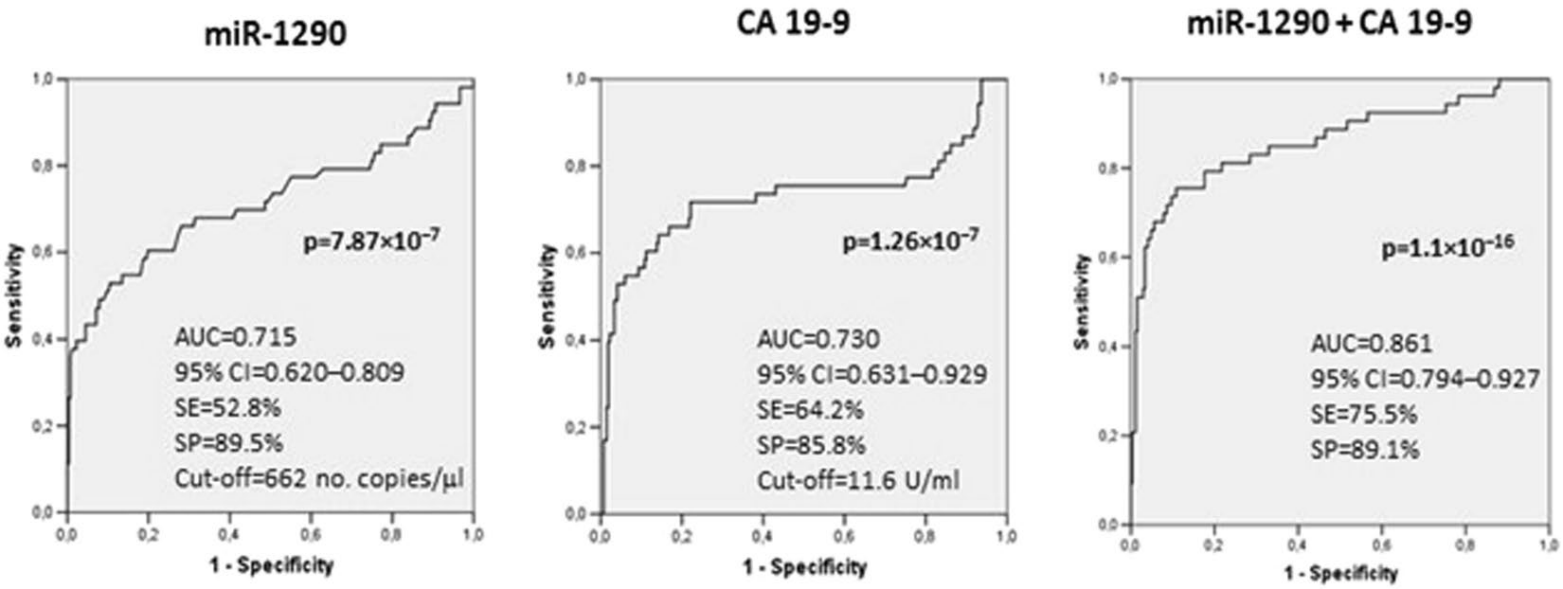
Receiver operating characteristics (ROC) curve analysis for discriminating healthy subjects (HS) from pancreatic cancer (PC) patients with slightly increased CA 19-9 levels. The diagnostic performance of miR-1290 and CA 19-9 was tested independently and then in combination using a logistic regression model.

**Figure 4:**
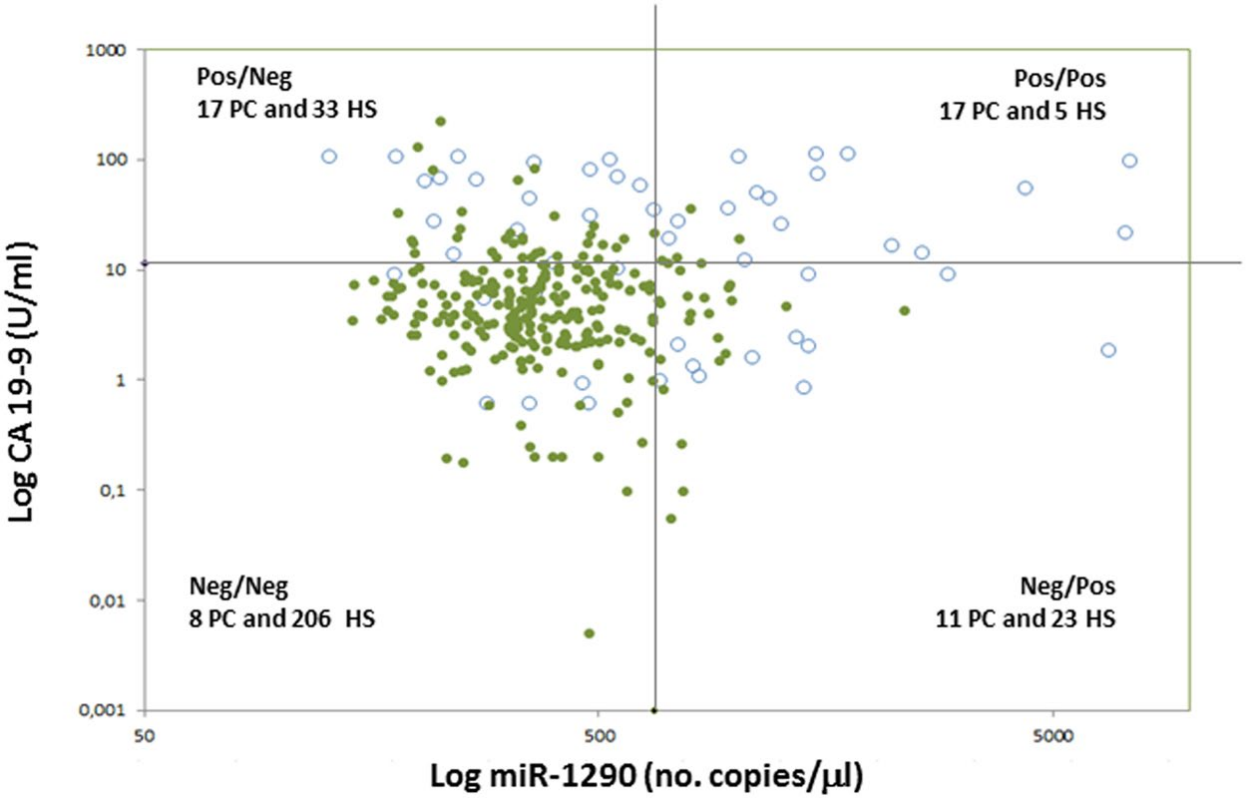
Scatter plotting of miR-1290 plasma levels against CA 19-9 serum value for healthy subjects (HS) and pancreatic cancer (PC) patients with slightly increased CA 19-9 levels. White circles: PC; green circles: HS; horizontal black line: cut-off value for CA 19-9 (11.6 U/ml) for differentiating HC from PC patients with slightly increased CA 19-9 levels; vertical black line: cut-off value for miR-1290 (662 no. copies/μl) for differentiating HC from PC patients with slightly increased CA 19-9 levels. Values are reported in log scale.

Next, we sought to ascertain the true contribution of miR-1290 to evaluation using CA 19-9 alone. To accomplish this, the entire cohort of PC patients was stratified according to individual CA 19-9 values: in 53 PC patients (15 with resectable, 17 with locally advanced, and 21 with metastatic disease) the CA 19-9 levels were normal or slightly increased (<3 times the upper normal value of 37 U/ml). In this subset of patients the regression model combining the two variables for discriminating PC patients from HS was more effective than evaluation based on either miR-1290 or CA 19-9 alone (Fig. 3). Furthermore, plotting the individual miR-1290 plasma levels against CA 19-9 serum values showed that 11 out of the 53 (20.8%) PC patients with normal or slightly increased serum CA 19-9 exhibited high miR-1290 values (Fig. 4). In details, only 6 out of these 11 PC patients were diagnosed with resectable PC, while the remaining 5 patients were affected by a locally advanced (n = 1) or a metastatic (n = 4) disease.

### Associations with clinical risk factors for PC development

In order to examine the association between miR-1290 plasma levels and the various risk factors for PC, both PC patients and HS were stratified into three subgroups according to number of factors. Data are shown in Table 2: miR-1290 plasma levels did not show significant differences between the low-, intermediate- and high-risk subgroups of PC patients and HS. The lack of statistical association persisted when the association with CA 19-9 serum levels was evaluated.

**Table 2:** Associations of clinical risk factors for pancreatic cancer (PC) with miR-1290 and CA 19-9 levels.

	PC (N = 159*)		miR-1290 (no. copies/μl)		p-value	CA 19-9 (U/ml)		p-value
	N	%	Median	Q1–Q3		Median	Q1–Q3	
Low risk	12	7.5	1158	710–1700	ns	413.05	30.265–1394	ns
Intermediate risk	114	71.7	672	352–1452		294.6	97.45–1709.1	
High risk	33	20.8	744	480–2524		400	63.86–1276	
	**HS (N** = **267)**		**miR-1290 (no. copies/μl)**			**CA 19-9 (U/ml)**		
	**N**	**%**	**Median**	**Q1–Q3**		**Median**	**Q1–Q3**	
Low risk	42	15.7	352	288–488	ns	4.2	2.5–7.6	ns
Intermediate risk	195	73.0	372	277.2–500		4.3	2.4–8.3	
High risk	30	11.3	344	269.6–500		5.5	3.2–8.9	

Low- Intermediate- and High-risk subgroups included subjects having 0–1, 2–3 and 4–6 risk factors emerged from the clinical investigation, respectively.

*PC patients with complete information on the six risk factors were included in the association analysis. HS: healthy subjects.

### Specificity of the plasma miR-1290 levels

**Figure 5:**
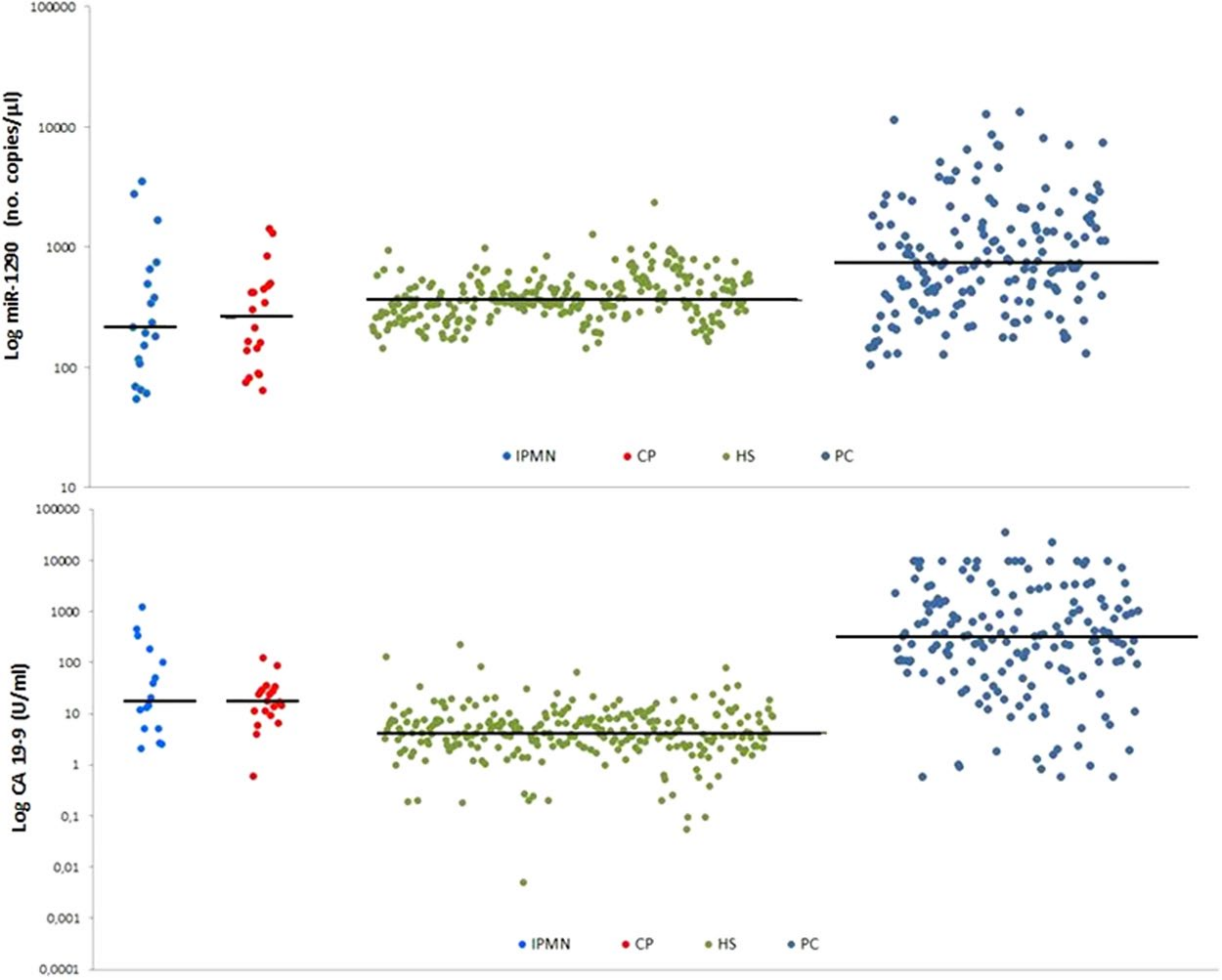
Absolute quantification of circulating levels of miR-1290 plasma and CA 19-9 in subjects with intraductal papillary mucinous neoplasm (IPMN) of the pancreas and chronic pancreatitis (CP) compared with healthy subjects (HS) and pancreatic cancer (PC) patients. Each blot indicates the levels of miR-1290 and CA 19-9 tested by droplet digital PCR and ELISA assay, respectively. The median levels in each subgroup of patients and in HS are indicated by the horizontal black bars. Values are reported in log scale (y-axis).

The distributions of miR-1290 and CA 19-9 levels in patients with IPMN and chronic pancreatitis (CP) are shown in Fig. 5.

Median circulating miR-1290 levels were 220 (Q1–Q3 = 108–664) no. copies/μl in IPMN, 257.8 (Q1–Q3 = 114.2–466) no. copies/μl in CP, and 360 (Q1–Q3 = 281.2–496) no. copies/μl in HS. These differences approached the level of significance when compared with the median values of HS (p = 0.05 and p = 0.04, respectively), but were significantly lower than the value seen in PC patients (p < 0.0001). As to CA 19-9 levels, their median values were significantly higher (p < 0.001) in patients with IPMN (17.9 U/ml; Q1–Q3 = 5.1–143.9) and CP (17.4 U/ml; Q1–Q3 = 10.1–28.5) compared with either HS (4.3 U/ml; Q1–Q3 = 2.4–7.9) or PC patients (299 U/ml; Q1–Q3 = 70.1–1615).

### Association of miR-1290 with pathological data in PC patients

Only PC aggressiveness was statistically correlated with circulating miR-1290 levels. All other considered clinical features (including tumor localization, tumor differentiation, tumor stage, surgical resection, and resection margins) were unrelated to miR-1290 plasma levels.

**Figure 6:**
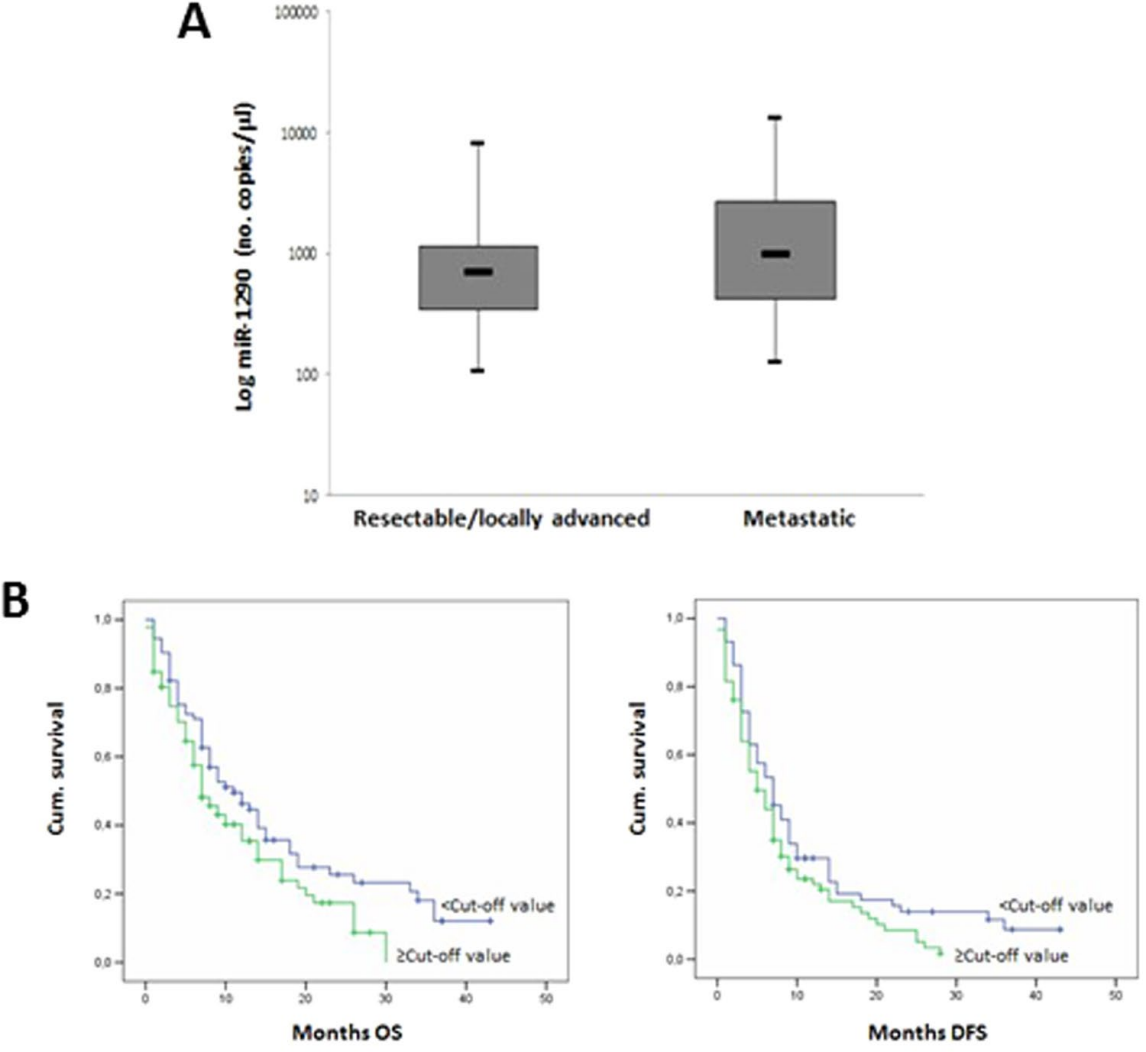
Association between miR-1290 plasma levels and pre-operative classification of pancreatic cancer (**A**). The box indicates median, interquartile range (Q1–Q3) and lower and upper adjacent values (vertical bars) for each subject group. miR-1290 values are reported in log scale (y-axis). Kaplan–Meier curve for survival (OS) and disease-free survival (DFS) in patients with pancreatic cancer according to the cut-off value of 662 n.copies/μl derived from the ROC curve for circulating miR-1290 levels (**B**). Cum.: cumulative.

As represented in Fig. 6A, miR-1290 plasma levels were significantly higher (p = 0.008) in patients preoperatively diagnosed as having metastatic PC (median value of 964 no. copies/μl; Q1–Q3 = 428–2756) compared with those with resectable or locally advanced disease (median value of 670 no. copies/μl; Q1–Q3 = 344–1142).

### Survival analysis

Univariate analysis showed that PC patients with miR-1290 plasma levels above the cut-off value of 662 n.copies/μl (derived from the ROC curve for circulating miR-1290) had shorter OS (p = 0.042) and DFS (p = 0.049) than those with miRNA plasma levels below the cut-off value (Fig. 6B). Data from the multivariate analysis of all characteristics with a prognostic impact (TNM classification and cancer staging, surgery, resectability and adjuvant chemotherapy) are shown in Supplementary Table 2: high miR-1290 plasma levels did not persisted as an independent negative prognostic factor for OS and DFS PC patients (p > 0.05).

## Discussion

CA 19-9 is the standard serum marker used in routine clinical practice for the diagnosis of PC and for monitoring its clinical course^5,6,21–24^. However, despite the sensitivity of CA 19-9 for detecting disease recurrence after surgery, its low specificity impairs its usefulness for early diagnosis of the disease^6^. Therefore, new and effective biomarkers are urgently needed to improve PC diagnosis and survival rates, and for use in screening programs for the early detection of small pancreatic lesions at a pre-invasive stage in high-risk asymptomatic individuals.

To date several studies have shown that circulating miRNA could be aberrantly expressed in PC patients^25^. Although quantitative PCR is the method of choice to quantify miRNA plasma levels, it has insufficient sensitivity^26^. However, ddPCR technology, which avoids some of the methodological problems of other molecular techniques, has recently facilitated the absolute quantification of miRNAs in body fluids^27–29^.

The usefulness of ddPCR for quantifying circulating miRNAs was tested in different human cancers, with the best diagnostic accuracy found for breast cancer^30,31^. There is limited information so far for PC, where the ddPCR methodology has been used mainly for the detection of KRAS mutations in circulating tumor DNA in early-stage PC patients^32–34^. In the present investigation, we sought to explore in more detail the diagnostic performance of ddPCR in assessing plasma levels of miR-1290 for discriminating HS from patients with PC, CP and IPMN, and to examine miR-1290 values in PC patients in relation to disease stage and prognosis. Overall a possible pitfall of our study in not having checked out the performance of miRNA isolation in different plasma samples. Although we started from an equal volume of plasma for all the PC patients and HS, the use of synthetic spike-in controls during the extraction process would have allowed to correct for possible differences in miRNA recovery efficiency among different samples.

As expected, we found miR-1290 plasma levels had good ability to discriminate between HS and PC in the exploratory cohort, which was confirmed in the validation one. Our findings are in agreement with the results of Ang *et al*. who used quantitative PCR^15,35,36^ to investigate the performance of both miR-1290 and CA 19-9 levels in HS and patients with PC. However, in contrast to the findings of those authors, we showed that evaluation of CA 19-9 levels had better discriminatory ability than miR-1290 measurement. In addition, it was only after combining the two tests that we could separate the cancer cohort from the HS cohort.

Elevated levels of circulating CA 19-9, in the appropriate context of clinical and radiological data, may signal a lesion within the pancreatic gland. However, the biomarker has low sensitivity as CA 19-9 can be falsely negative for some PC patients, including the 10% of subjects negative for Lewis antigens who do not synthesize CA 19-9^37,38^.

Overall our data in the entire cohort showed that CA 19-9 is significantly better compared to miR-1290 plasma levels, suggesting a lack of clinical utility for the miRNA in distinguishing PC patients from HS. However, grouping PC patients according to individual levels of circulating CA 19-9 resulted in a novel observation: of 53 PC patients with normal or slightly elevated (<3 times the upper normal limit of 37 U/ml) concentrations of the serum marker CA 19-9, 11 had elevated levels of circulating miR-1290. Thus, limited to patients with little increase in the conventional marker, this finding suggests that concurrent evaluation of the two biomarkers could increase their sensitivity for identifying a cancer lesion within the pancreas.

The specificity of the CA 19-9 assay for ruling out PC is also uncertain, as serum levels of the biomarker may also increase in inflammatory conditions of the biliary tree or the pancreas. We tried to address this point by including in our investigated population 39 subjects with non-cancerous pancreatic lesions: half of these patients had inflammatory disease of the pancreas (CP), and half pre-cancerous pancreatic lesions (IPMN). Although CA 19-9 levels in the latter group were significantly above the mean value observed in HS, miR-1290 levels were normal. In addition, miR-1290 concentrations were unable to differentiate among PC patients with single or multiple risk factors for PC. All in all, these results suggest that miR-1290 levels cannot be used to select patients at clinical risk for PC for entry into a surveillance program.

A final aim of the present investigations was to correlate miR-1290 levels with the pathological characteristics of PC, and with outcome in surgically resected patients. In the overall cancer population, higher miR-1290 levels were more often present in patients with metastatic disease, suggesting cancer aggressiveness and/or tumor burden are major determinants of serum concentrations of the biomarker. Higher miR-1290 levels were also associated with poorer OS and DFS rates in PC patients, although not as an independent negative prognostic factor. Despite the potential of ddPCR for the absolute quantification of circulating miRNA levels, our study has failed to confirm circulating miR-1290 levels as a new diagnostic biomarker for PC. Further evaluations are required to advance the unmet clinical need for new PC biomarkers differentiating subjects with pancreatic disease from healthy individuals, and malignant from benign pre-cancerous lesions of the pancreas.

## Material and Methods

### Study population and subject enrollment

This collaborative prospective study was conducted at the Divisions of Gastroenterology, Surgery, and Oncology of “Casa Sollievo della Sofferenza” Hospital, San Giovanni Rotondo, Italy between November 2011 and May 2016. A total of 473 subjects were enrolled. Of these, 167 had a final diagnosis of PC based on histology (n = 132) or imaging (n = 35). In addition, 267 individuals were HS attending the outpatient clinic of the Division of Gastroenterology for digestive complaints; those with a final diagnosis of functional gastrointestinal disease (e.g., gastritis, gastro-esophageal reflux disease, dyspepsia, irritable bowel syndrome and somatic stress) were retained into the study after a clinical evaluation and ultrasound imaging disclosed a normal pancreas. Furthermore, all these patients were followed with interview at 1 and 3 years after enrollment.

All PC patients and HS were inquired about the occurrence of factors predisposing to PC, namely age ≥55 years, diabetes, smoking, alcohol abuse, high body mass index, and a family history of PC or any cancer.

Two smaller groups were used as internal cohorts of patients with pre-tumoral or inflammatory pancreatic disease: one group consisted of 19 patients with IPMN, and the other of 20 patients with CP.

Before enrolment into the study, all subjects signed an informed consent form after the aims of the study had been to them explained by a research nurse. The Ethics Committee of the IRCCS Hospital “Casa Sollievo della Sofferenza” approved the study (Prot. No. 96/CE/2011) which was conducted in accordance with approved guidelines.

Blood samples were then taken from all subjects for the determination of miR-1290 and CA 19-9 levels. Samples from PC patient who were undergoing pancreatic resection were collected before surgery, whereas for patients with locally advanced or metastatic disease, samples were obtained at diagnosis and before any chemotherapy had been initiated.

### Sample collection and miRNA purification

Blood samples were collected in BD Vacutainer™ Plus Plastic Citrate Tubes and processed within 1 h. Tubes were centrifuged at 3500 rpm for 10 min at room temperature, and plasma was stored at −80 °C until use. Total RNA, including the miRNA fraction and other small RNAs (<200 nt), was isolated from 100 μl of plasma by using the MiRNeasy kit (Qiagen, Hilden, Germany; cat. no. 217004), according to the manufactures’ instructions.

In order to adjust for differences in recovery efficiency between different plasma samples, we used equal volume of starting material for all the plasma samples, as it is advisable to have accurate results for biomarker detection study^39^.

### Assessment of haemolysis

To limit the impact of the haemolysis on miRNAs content in plasma^40^, we followed standard guidelines together with the SOPs for plasma samples collection and preparation for miRNA evaluation^41,42^. Visible hemolyzed sample (suggested by orange to pink and dark pink to red discoloring) have been excluded for analysis. In addition, the low-grade haemolysis was assessed by spectrophotometry (NanoDrop™ 1000, Thermo Fisher Scientific, Inc., Waltham, MA, USA). Samples were classified as hemolyzed or not-hemolyzed based on the optical density of free hemoglobin at 414 nm, as previously reported^43^. In addition, a lipemia-independent haemolysis score was also calculated based on absorbance measurements at λ = 385 nm^44^. Since altered miRNA levels in PC patients might be due to other cancer-associated conditions rather than to a real effect of haemolysis on miRNA plasma levels, both the absorbance at 414 nm and the lipemia- independent haemolysis score were computed for HS and PC patients, separately.

### Reverse transcription

cDNA was synthesized starting from 1 μl of extracted RNA using the TaqMan miRNA Reverse Transcription Kit and miRNA-specific stem-loop primers (Applied BioSystems, Foster City, CA; cat. No. 4427975, assay ID 002863) following manufacturer’s instructions.

In order to normalized the sample-to-sample variability in miRNA content, we used a fixed volume of eluted RNA sample as input, rather than the same amount of RNA for each sample. Indeed, the yield of RNA from small volume of plasma/serum has been reported to be below the limit of accurate quantitation by spectrophotometry, thus miRNA contents extracted from plasma/serum has been reported to be undetectable by using NanoDrop spectrophotometer^42,45,46^. In addition, it should be considered that cancer may cause a release of nucleic acids in the circulation, leading to a significant higher level of circulating RNA in cancer patients than in healthy subjects^47,48^.

### Droplet digital PCR

Total miR-1290 plasma levels were then quantified using the ddPCR system (Bio-Rad Laboratories, Hercules, CA). Briefly, 5 μl of the synthesized cDNA were added to a 20 μl PCR reaction mixture containing 10 μl of digital PCR™ supermix (Bio-Rad Laboratories), 1 μl of TaqMan primer/probe mix (Applied BioSystems) and RNase-free H_2_O. Droplets were generated by loading the mixture into a plastic cartridge with 70 μl of QX100 Droplet Generation oil (Bio-Rad Laboratories). Cartridges were then placed into the QX200 Droplet Generator (Bio-Rad Laboratories). The droplets generated from each sample were transferred to a 96-well PCR plate (Eppendorf, Hamburg, Germany), and PCR amplification was carried out on the C1000 Touch Thermal Cycler (Bio-Rad Laboratories), according to the manufacturer’s protocol. The plate was then loaded on the QX200 Droplet Reader (Bio-Rad Laboratories) and read automatically. The fraction of PCR-positive droplets was quantified assuming a Poisson distribution^49^. In details, the QuantaSoft software was used to obtain the concentration results in no. copies per microliter (no. copies/μl) for each sample. Then, in order to convert these results in the no. copies present in the starting sample, the above concentration values were multiplied for the reaction volume, and divided for the starting volume of cDNA.

### Serum CA 19-9 determination

Blood was collected in BD Vacutainer® Serum separating Tube (SST), and the serum was separated by centrifuged at 3000 rpm for 10 minutes and frozen at −80 °C until assayed. CA 19-9 serum levels were determined by using a commercially available CA 19-9 enzyme immunoassay (TM-CA 19-9 ELISA, cat. no. EIA-5069; DRG Instruments, Marburg, Germany), according to the manufacturer’s instructions. Determinations were carried out in all the HS samples, whereas the serum CA 19-9 value for PC patients were recovered from the clinical records. In order to exclude differences in CA 19-9 levels due to different quantification methods between HS and PC patients, the CA 19-9 was also randomly determined in a subset of PC serum samples. As expected, all the value obtained by the enzyme immunoassay corresponded to those recovered from the clinical records.

### Statistical analysis

Continuous and categorical variables were reported as medians with interquartile ranges and absolute and relative frequencies, respectively, and compared using a non-parametric 2(k)-independent sample test according to the Mann–Whitney, Kruskal–Wallis, Pearson χ^2^ or Fisher’s exact test, where appropriate.

To estimate the ability of miR-1290 and CA 19-9, and their combination, to discriminate HS from PC patients, the entire study population was randomly divided into a discovery and a validation cohort, according to the time of their inclusion into the study. The former cohort included PC patients and HS enrolled between November 2011 and June 2013, and the latter those enrolled between October 2013 and May 2016.

The best discriminating cut-off values for miR-1290 and CA 19-9 levels were derived from the receiver operating characteristic (ROC) curves, which were first drawn separately for each of the two cohorts. Results from the two cohorts were then pooled and global diagnostic accuracy was evaluated. Finally, miR-1290 and CA 19-9 levels were combined using a binary logistic regression model, and the best model’s cut-off value for discriminating HS from PC patients was investigated for each experimental cohort and for the entire enrolled population.

The Kaplan–Meier method was used to estimate the distribution of overall survival (OS) and disease-free survival (DFS) for PC patients. The effect of several risk factors on the clinical outcomes of PC patients was evaluated by univariate analysis with the log rank test. Significant variables at univariate analysis were entered into a forward multivariate Cox’s regression analysis to identify the independent prognostic factors for PC. The results were expressed in terms of hazard ratios (HR) and 95% confidence intervals (95% CI).

Statistical analysis was performed using the SPSS software package v. 13.0 (Chicago, IL, USA) and statistical significance was set at p < 0.05.

## Electronic supplementary material

Supplementary Tables
