# Supplementary material for: Droplet digital PCR quantification of miR-1290 as a circulating biomarker for pancreatic cancer
Source: Sci Rep. 2018 Nov 6;8:16389. doi: 10.1038/s41598-018-34597-z (PMC6219528; doi:10.1038/s41598-018-34597-z)
Supplement: Supplementary file 1 — Supplementary Tables [file 41598_2018_34597_MOESM1_ESM.pdf]

Droplet digital PCR quantification of miR-1290 as a circulating biomarker for pancreatic cancer

Francesca Tavano[1*], Domenica Gioffreda[1], Maria R. Valvano[1], Orazio Palmieri[1],Matteo Tardio[2], Tiziana P. Latiano[3], Ada Piepoli[1], Evaristo Maiello[3], Felice Pirozzi[2], Angelo Andriulli[1]

Division of Gastroenterology and Research Laboratory[1], Department of Surgery[2], Department of Oncology[3], "Casa Sollievo della Sofferenza" Hospital, I.R.C.C.S., Viale Cappuccini 1, San Giovanni Rotondo (FG), 71013, Italy

* Corresponding author

Table S1. Demographics and distribution of clinical risk factors in healthy subjects (HS) and patients with pancreatic cancer (PC), and pathological characteristics of PC patients divided into discovery and validation cohorts						
	HS			PC		
	Discovery cohort (N=150)	Validation cohort (N=117)	p-Value	Discovery cohort (N=93)	Validation cohort (N=74)	p-Value
**Age, median (Q1–Q3)**	60 (54–67)	62 (55–70)	ns	68 (60–76)	69 (61–76)	ns
**<55 years, N (%)**	41 (27.3)	29 (24.8)		12 (12.9)	11 (14.9)	
**≥55 years, N (%)**	109 (72.7)	88 (75.2)	ns	81 (87.1)	63 (85.1)	ns
**Gender, N (%)**						
**Male**	63 (42)	53 (45.3)		46 (49.5)	40 (54.1)	
**Female**	87 (58)	64 (54.7)	ns	47 (50.5)	34 (45.9)	ns
**Body Mass Index, median (Q1–Q3)**	26 (24–30)	28 (25–30)	ns	25 (22–28)	25 (23–28)	ns
**≤30, n (%)**	114 (76)	90 (76.9)		75 (83.3)	63 (87.5)	
**>30, n (%)**	36 (24)	27 (23.1)	ns	15 (16.7)	9 (12.5)	ns
**Smoker, N (%)**						
**No**	68 (45.3)	65 (55.6)		43 (46.2)	28 (37.2)	
**Current/past**	82 (54.7)	52 (44.4)	ns	50 (53.8)	46 (62.2)	ns
**Alcohol abuse (≥3 drinks a day), N**						
**No**	142 (94.7)	108 (92.3)		85 (92.4)	66 (89.2)	
**Yes**	8 (5.3)	9 (7.7)	ns	7 (7.6)	8 (10.8)	ns
**Diabetes, n (%)**						
**No**	126 (86)	101 (86.3)		61 (65.6)	41 (55.4)	
**Yes**	21 (14)	16 (13.7)	ns	32 (34.4)	33 (44.6)	ns
**Family history of cancer, N (%)**						
**No**	42 (28)	28 (24.1)		30 (33.7)	27 (37.5)	
**Yes**	108 (72)	88 (75.9)	ns	59 (66.3)	45 (62.5)	ns
**Tumor location, N (%)**						
**Head**				68 (74)	49 (67)	
**Body**				12 (13)	9 (12)	
**Tail**				12 (13)	15 (21)	ns
**Pre-operative classification, N (%)**						
**Resectable**				18 (19)	16 (22)	
**Locally advanced**				35 (38)	25 (34)	
**Metastatic**				40 (43)	33 (44)	ns
**Surgery, N (%)**						
**No**				80 (86)	58 (78)	
**Yes**				13 (14)	16 (22)	ns
**Tumor stage, N (%)**						
**IB**				1 (1.1)	–	
**IIA**				1 (1.1)	3 (4.2)	
**IIB**				14 (15.9)	11 (15.5)	
**III**				32 (36.4)	24 (33.8)	
**IV**				40 (45.5)	33 (46.5)	ns
**Adjuvant therapy, N (%)**						
**No**				36 (40)	27 (37)	
**Yes**				54 (60)	46 (63)	ns

Table S2. Multivariate analysis for overall survival (OS) and disease-free survival (DFS)

		OS			DFS		
	**Variable**	**HR**	**95% CI**	**p-Value**	**HR**	**95% CI**	**p-Value**
**miR-1290**	≥ 662 no.copie/μl	–	–	0.140	–	–	0.09
**Distant metastasis status***	M1	1.764	1.032-3.016	0.04	–	–	0.487
**Adjuvant therapy**	No	3.301	2.01-5.422	<0.0001	1.582	1.047-2.393	0.03
**Pre-operative classification**	Locally advanced	2.109	1.118-3.979	0.02	1.696	1.034-2.782	0.04
**Primary tumor status***	T4	–	–	0.669	–	–	0.533
**Surgery**	No	–	–	0.583	–	–	0.268

*According to the AJCC tumor/nodes/ metastasis (TNM) classification for pancreatic cancer [21]
